# Comment on Krysik et al. Clinical Outcomes and Early Postoperative Complications in Boston Type I Keratoprosthesis Implantation: A Retrospective Study. *J. Clin. Med.* 2024, *13*, 6710

**DOI:** 10.3390/jcm14051551

**Published:** 2025-02-26

**Authors:** David Mikhail, Daniel Milad, Mona Harissi-Dagher

**Affiliations:** 1 Temerty Faculty of Medicine, University of Toronto, Toronto, ON M5S 3K3, Canada; d.mikhail@mail.utoronto.ca; 2 Department of Ophthalmology, University of Montreal, Montreal, QC H3T 1J4, Canada; 3 Department of Ophthalmology, Centre Hospitalier de l’Université de Montréal (CHUM), Montreal, QC H2X 3E4, Canada

We read with great interest the article “Clinical Outcomes and Early Postoperative Complications in Boston Type I Keratoprosthesis Implantation: A Retrospective Study” by Krysik et al. [1]. We commend the authors for their comprehensive assessment of early postoperative complications associated with Boston Type I Keratoprosthesis (BKPro) implantation and their insights into the challenges of managing these complex cases.

The study reports an incidence of glaucoma in 32 eyes (42%), retroprosthetic membrane (RM) formation in 20 eyes (26%), and BKPro extrusion in 10 eyes (13%) among 77 eyes undergoing BKPro implantation. These findings align with previously reported rates of postoperative complications.

Glaucoma emerged as the most common complication. The authors appropriately underscore the importance of intraocular pressure (IOP) control, treating glaucoma postoperatively with laser cyclophotocoagulation, trabeculectomy, and topical therapy. Nonetheless, glaucoma progression and visual decline remain significant challenges, with visual acuity (VA) reduced to light perception in 7.8% of patients.

We recommend that glaucoma surgery be performed prior to or concurrently with BKPro implantation in eyes with preexisting glaucoma to slow the progression of optic nerve cupping [2]. The authors’ findings aligned with this recommendation, as preoperative surgical interventions (e.g., Ahmed glaucoma valve and peripheral iridectomy) reduced reliance on pharmacological IOP management, eliminating the need for additional postoperative glaucoma surgery. However, even with careful IOP control, our group has observed continued glaucoma progression, which may be attributed to higher scleral rigidity and altered biomechanical forces introduced by BKPro’s polymethyl methacrylate optic and backplate [3].

Our group has previously found that high preoperative IOP significantly predicts both de novo glaucoma development (OR = 1.538, 95% CI: 1.030–2.297; *p* = 0.035) and progression (OR = 1.450, 95% CI: 1.084–1.937; *p* = 0.012) [4]. In this study, detailed analysis of IOP values and methods of pressure measurement before and after implantation could have further strengthened the discussion of glaucoma risk factors. Additionally, it would be valuable to distinguish between outcomes in eyes with preexisting glaucoma and those developing glaucoma after BKPro implantation, particularly regarding differences in IOP, VA, and treatment efficacy.

The authors report that 36% of patients with severe corneal surface damage had autoimmune diseases. Evaluating the glaucoma status of these patients is of particular interest, since autoimmune conditions and ocular surface diseases have been linked to the earlier onset of de novo glaucoma [4]. Stratifying outcomes of autoimmune diseases could further clarify mechanisms driving postoperative complications. Stevens–Johnson syndrome, for example, has been associated with a sevenfold increased risk of vision loss following BKPro implantation (HR = 7.3, 95% CI: 2.5–21.5; *p* < 0.001), highlighting the need for rigorous preoperative evaluation and tailored management strategies for these high-risk patients [5].

In conclusion, this study effectively delineates the early postoperative complications of BKPro implantation, reinforcing the importance of comprehensive preoperative planning, including IOP control and optimization of ocular surface health, along with vigilant postoperative management to address vision-threatening complications like glaucoma.
